# Comments to Article by Thoene M et al., *Nutrients* 2016, *8*, 451

**DOI:** 10.3390/nu8120821

**Published:** 2016-12-16

**Authors:** Fernando Moya

**Affiliations:** Betty Cameron Children’s Hospital, 2131 S 17th St, Wilmington, NC 28401, USA; fernando.moya@ccneo.net

To the Editor:

I read with interest recent work published in your journal in July 2016 by Thoene et al. [1]. These authors attempted to address which human milk fortifier (HMF) is the optimal choice for preterm infants in a neonatal intensive care unit (NICU). To do this they reported again data from a previous retrospective study published in *Nutrients* on January 2014 [2] using a powdered HMF (P-HMF) with an acidified liquid HMF (AL-HMF), and compared it with additional retrospective data using yet a different non-acidified liquid HMF (NAL-HMF). The authors focused on relevant issues for neonatologists such as growth, impact of HMF’s in acid-base balance and the occurrence of necrotizing enterocolitis (NEC).

Whenever readers attempt to compare studies and validate conclusions, it becomes of utmost importance to understand the populations enrolled in such studies and how comparable was the intervention(s) of interest. Both retrospective studies by Thoene [1,2] selected a population of larger preterm infants without excluding for acuity. Thus, they reported a vastly different population than in the original prospective randomized trials by Moya et al. [3] evaluating the AL-HMF, which enrolled infants less than 30 3/7 weeks of gestation and <1250 g at birth, and the trial by Kim et al. [4] examining the NAL-HMF, which enrolled infants <33 weeks and range 700–1500 g at birth. Not surprisingly, the populations enrolled in these randomized trials were between 1–3 weeks younger in gestational age and about 200–400 g lighter in birth weight than in the retrospective studies of Thoene. Moreover, even though exclusion criteria differed between these randomized trials, both aimed to exclude infants with low Apgar scores and in need of either high inspired oxygen concentrations [3] or ventilatory support [4]. These trials described in detail relevant demographic characteristics of their populations such as proportion of infants <1000 g, mode of delivery, Apgar scores, and provided descriptive statistics of the range of gestational age and birth weight. Much of this information is missing in both reports by Thoene, including ranges or standard deviations for many continuous variables, especially for the new retrospective group of infants receiving the NAL-HMF from their most recent study [2]. Moreover, there are multiple additional discrepancies in data between both studies by Thoene, which make it harder to reconcile their findings and conclude how representative is their population, i.e., “N” for AL-HMF group for gestational age, birth weight, length and head circumference at 36 weeks, death, number of infants with bronchoplumonary dysplasia. This is also the case for other data, such as day of life when feedings were started, “N” for CO_2_ minimum on day 14 and 30 (Tables 3 and 4 from their first and follow up reports, respectively). As mentioned before, without either medians with interquartiles, or means with standard deviations, one cannot get a more accurate overview of the distribution of relevant findings such as CO_2_ values and rate of growth, for instance.

Indeed the issue of metabolic acidosis, whatever such definition might be in preterm newborns, is relevant and its clinical importance has been brought up regarding the use of HMF’s. In our original blind randomized trial comparing P-HMF with AL-HMF, there was a decrease in CO_2_ particularly on day 14 after starting AL-HMF [3]. This notwithstanding, the 25% interquartile was at a level of 20 mEq/L meaning that very few infants had CO_2_ levels below that threshold. Moreover, none of the 146 infants enrolled (of which 74 received AL-HMF) was given treatment for metabolic acidosis as judged by attending neonatologists blind to the intervention. Furthermore, both groups of infants exhibited mean rates of growth of about 15–16 g/kg/day, well above what Thoene reported for the retrospective AL-HMF group [1]. Besides the studies by Thoene, in another retrospective study Cibulskis and Armbrecht [5] reported a high proportion of infants developing metabolic acidosis while using the AL-HMF compared to a historical group exposed to P-HMF. These authors defined metabolic acidosis as base deficit greater than 4 mmol/L or serum bicarbonate <18 mmol/L. Both articles by Thoene do not provide a definition of metabolic acidosis, even though from the only data providing ranges (Figures 1 and 2 of *Nutrients* 2014 article) the proportion of infants with CO_2_ values <18 was clearly higher in the AL-HMF group. These authors associated this metabolic acidosis to a much slower rate of growth with AL-HMF (10.59 g/kg/day), thereby ascribing clinical significance to such finding. This was not corroborated in the report by Cibulskis [5] showing similar rates of growth between fortifiers, which were actually higher than those reported by Thoene for a somewhat similar population of infants and approximated those reported in the Moya trial [1,2,3]. Moreover, in a recent prospective, randomized trial of timing of human milk fortification by Shah et al. [6], in which the only fortifier used was AL-HMF, there was an overall 7% rate of metabolic acidosis using a far more liberal definition (base deficit ≥10 mEq/L) and the rate of growth reported at 36 weeks postmenstrual age was higher than in the Thoene and Cibulskis reports. What might explain these differences other than selection bias? In the trial by Shah, all infants received either mother’s milk or donor milk (about two thirds of infants) until reaching either 1500 g or 34 weeks of gestation; those infants getting donor milk were then switched to preterm formula. Growth rate in all infants was calculated at 4 weeks of age and at 36 weeks [6]. In both studies by Thoene [1,2] and Cibulskis [5], it is unclear what proportion got donor milk and for how long. In addition growth rates were calculated by Thoene only among infants while they received >50% of fortified mother’s milk, but we do not know at all what proportion of the remaining nutrition came from donor milk or formula except for a brief statement in the discussion mentioning that formula was utilized equally in all groups [2]. Use of formula is a known risk factor for the development of NEC.

Finally there is the issue of NEC. Thoene makes strong statements about this severe complication of prematurity based on an extraordinarily low number of subjects (3 of 23 in AL-HMF group), and more importantly, without even defining how was NEC diagnosed. All other studies referenced here reported lower rates of this complication and used a stricter and widely accepted definition of proven/confirmed NEC [3,4,5,6]. This is crucial, as milder forms of suspected NEC overlap clinically with signs of feeding intolerance, a very common problem among infants receiving HMF’s [3,4,5,6]. Determining whether use of HMF’s in general, or anyone of them in particular, is associated with NEC is quite important. 

In conclusion, the use of HMF’s is critical for optimal nutrition of preterm infants. This intervention, like many others provided to a susceptible population, may be associated with unexpected and/or clinically significant side effects. Looking forward, the best evidence as to which of the available liquid HMF’s is the most optimal choice for premature human-milk fed infants can only come from well-designed, prospective, randomized (and ideally blind) trials, in which some of the aforementioned limitations of retrospective data discussed in this comment, can be avoided or better controlled. Moreover, in spite of the fact that the Moya trial is the largest randomized trial of liquid HMF’s of bovine origin [3], it was not powered to examine the issue of NEC. The neonatology community would benefit immensely if future comparison trials of HMF’s are also powered to provide a more definitive answer to this conundrum.
